# Assessment of Knowledge, Attitude, and Practice Regarding Diabetic Retinopathy in an Urban Population in Northeast China

**DOI:** 10.3389/fpubh.2022.808988

**Published:** 2022-03-11

**Authors:** Jin-Yan Qi, Gang Zhai, Yu Wang, Yuan-Bo Liang, Dong Li, Liang Wen, Dong-Xiao Zang, Ke-Mi Feng, Bo Zang, Cong Xie

**Affiliations:** ^1^Fushun Eye Hospital, Fushun, China; ^2^School of Ophthalmology and Optometry, Wenzhou Medical University, Eye Hospital of Wenzhou Medical University, Wenzhou, China

**Keywords:** Northeast China, urban population, knowledge, attitude, practice

## Abstract

**Objective:**

This study aimed to assess the knowledge, attitude, and practice (KAP) of diabetic subjects with diabetic retinopathy (DR) and those without DR (NDR) in an urban community in Northeast China, as well as their risk factors in subjects with DR and NDR.

**Methods:**

A community-based survey involving 1,662 subjects was conducted in Fushun, China, between July 2012 and May 2013. The subjects included diabetics with DR (*n* = 783) and those NDR (*n* = 879), and questionnaires were completed to collect information about their sociodemographic and healthcare characteristics. A Chi-square test and multiple logistic analyses were performed to analyze the data.

**Results:**

Among the DR group, 21.88% had a good knowledge of DR, 94.15% had a positive attitude, and 68.07% followed good practice, whereas 20.98% of the NDR group had a good knowledge of DR, 94.18% had a positive attitude, and 66.92% followed good practice. There was no significant difference in the KAP of the two groups of subjects. In the NDR group, a good level of knowledge was associated with a high-level of education (OR = 0.1, 0.2; *p* < 0.05), a good attitude was associated with retirement (OR = 0.2; *p* < 0.05), and good practice was associated with being female, having a high-level of education, and the type of treatment (OR = 0.5, 0.4, 2.3, 3.1; *p* < 0.05). In the DR group, good practice was associated with older age and retirement (OR = 0.6, 0.4; *p* < 0.05).

**Conclusions:**

There was no significant difference between the DR and NDR subjects in the overall levels of KAP, but both groups showed a poor level of knowledge. Age, gender, education, occupation, and type of treatment were the main factors associated with the KAP scores, more risk factors in the NDR group than in the DR group. There is an urgent need for coordinated educational campaigns with a prioritized focus on the northeast region of China, especially NDR group.

## Introduction

Diabetic retinopathy (DR) is a common severe microvascular complication in patients with diabetes, posing a heavy socioeconomic burden on individuals, communities, and countries (1). The management of DR largely depends on patients' ability to self-care in their daily lives. Knowledge, attitude, and practice (KAP) surveys are effective in providing information for evaluating intervention programs, and a patient's KAP are always considered essential elements of DR management. However, there is a paucity of data regarding KAP based on disease status (with DR and without DR) in Northern Cities of China (2). Knowledge refers to the understanding of DR, attitude refers to patients' preconceived ideas that may have an impact on DR, and practice refers to the control diabetics have over their state of health and any eye disorder. This study was designed to investigate the diabetes related KAP of diabetics with and without DR in an urban area of the northeast of China to identify possible ways of improving eye care as part of overall diabetes care.

## Methods

This community-based study was conducted between July 2012 and May 2013. The study adhered to the Declaration of Helsinki and ethics approval was obtained from the Fushun Eye Hospital. The inclusion criteria were that patients were above 30 years of age with a clinical diagnosis of Type 2 diabetes mellitus and medical records of their diagnosis and treatment. The only exclusion criterion was having a cognitive impairment.

Recruit all patients diagnosed with type 2 diabetes in the community through the health records managed by the community health centers, chronic disease management files and household surveys conducted by members of the project team in collaboration with the staff of the 15 community committees in General Street.

### The Measurement and Definitions of Covariates

After a thorough review of the literature, a suitably designed and validated KAP questionnaire was administered by trained investigators (clinicians and nurses). The questions were translated into Chinese, and the questionnaire was piloted in face-to-face interviews. The overall study protocol involved a systematic two-step evaluation. The first step involved a comprehensive eye examination and a basic systemic evaluation. The data collected included demographic details (age, gender, occupation, education, and whether or not they lived alone), the status of the subject's systemic condition (current treatment and disease duration), and a fundus photograph. The second step involved the completion of a 17-item KAP questionnaire [knowledge (7 items), attitude (5 items), and practice (5 items)]. The knowledge questions had four possible responses (completely aware, aware, don't know, and unaware), as did the attitude questions (extremely concerned, concerned, indifferent, and unconcerned) and the practice questions (always, often, sometimes, and rarely). Each KAP question scored from 4 to 1 in the same order as the responses above. Thus, the minimum and maximum scores were between 7 and 28, 5 and 20, and 5 and 20 for knowledge, attitude, and practice, respectively. A total score of more than 55% was taken as “good”, while a total score of <55% was considered “poor”. Subjects who could neither read nor write were assisted by a research assistant.

### Diabetic Retinopathy Assessments

Each subject underwent fundus photography following a standardized protocol that is commonly used in epidemiologic studies. After pupil dilation, six fields of color fundus photography, with stereoscopic macula images of each subject, were taken by certified photographers using a 45° non-mydriatic retinal camera (Kowa, VK-2, Tokyo, Japan). The six fields were as follows: Field 1, center of the optic disc; Field 2, center of the macula; Field 3, temporal to macula; Field 4, temporal superior; Field 5, temporal inferior; and Field 6, nasal to the optic disc. The photographs were all read by two trained ophthalmologists, and any inconsistencies in their findings were settled by a senior ophthalmologist. Fundus photographs were graded in a masked manner according to the modified Airlie House Classification system (3). The levels of retinopathy and ME were based on the grading of the worse eye. If an eye was unable to grade, the scores for the other eye were used. Eyes were graded according to the following criteria: (1) no DR (levels 10–20); (2) non-proliferative DR [mild (levels 31–37), moderate (levels 43–47), or severe (levels 53)] or (3) proliferative DR (levels 60–85). The diagnosis for each subject was based on the field/lesion with the highest stage of DR.

### Statistical Analysis

All statistical analyses were conducted using SPSS version 23. Descriptive statistics such as percentages, frequencies, and mean ± standard deviation were calculated. A Chi-square test was used to determine the associations between KAP and the DR and NDR groups. Multivariate logistic regression model was done to determine the relationship between risk factors and the DR and NDR groups. Statistical testing was with a significance level set at 0.05.

## Results

A total of 1,662 subjects (990 females and 672 males) with a mean age of 61.87 ± 8.63 years were evaluated, and the mean diabetes duration was 7.99 ± 5.94 years. The demographic characteristics of the two groups of subjects, based on their disease status (DR or NDR), are given in Table 1. It was found that 21.88% of the DR subjects had a good knowledge of DR, 94.15% had a good attitude, and 68.07% followed good practice, while among the NDR group 20.98% had a good knowledge of DR, 94.18% had a good attitude, and 66.92% followed good practice. There were no significant differences between the two groups in the “good” KAP scores.

**Table 1:** The demographic characteristics of NDR and DR.

	**NDR (%)**	**DR (%)**	***p*-value**
	***N* = 879**	***N* = 783**	
Age (years)			0.028
≤ 60	365 (41.52)	367 (46.87)	
>60	514 (58.48)	416 (53.13)	
Gender			0.289
Male	366 (41.64)	306 (39.08)	
Female	513 (58.36)	477 (60.92)	
Occupation			0.232
Employment	101 (11.49)	87 (11.11)	
Unemployment	52 (5.92)	63 (8.05)	
Retirement	726 (82.59)	633 (80.84)	
Education			0.071
Primary	524 (59.61)	504 (64.37)	
Middle	227 (25.82)	172 (21.97)	
High	128 (14.56)	107 (13.67)	
Living alone			0.331
Yes	91 (10.35)	70 (8.94)	
No	788 (89.65)	713 (91.06)	
Type of treatment			0.000
Oral pills	646 (73.49)	453 (57.85)	
Insulin	140 (15.93)	303 (38.70)	
Diet control	93 (10.58)	27 (3.45)	
Disease duration (years)	5.76 ± 4.70	10.50 ± 6.19	0.000
<10	702 (79.86)	344 (43.93)	
≥10	177 (20.14)	439 (56.07)	

*P < 0.05 for χ^2^ tests*.

*NDR, no diabetic retinopathy; DR, diabetic retinopathy*.

### Knowledge

Nearly one fifth of the subjects in both groups (21.88% DR and 20.98% NDR) were aware of the symptoms of DR, but only a minority (<7% in both groups) had a good knowledge of the cause, diagnosis, and treatment of DR. Over 80% of the subjects in both groups were aware that blood glucose control effectively delays the progression of DR. The odds of NDR subjects with a primary and middle level education having a good knowledge of DR were 0.1 (0.0, 0.2) and 0.2 (0.1, 0.6) times the odds for those with a high level of education, while other factors were not significantly associated with a knowledge of DR in either group (see Table 2).

**Table 2:** Multivariate logistic regression of good knowledge in NDR and DR.

	**NDR (*****N*** **=** **879)**				**DR (*****N*** **=** **783)**			
	**Good**	**Poor**	**OR (95%CI)**	***p*-value**	**Good**	**Poor**	**OR (95%CI)**	***p*-value**
**Age (years)**
≤ 60	7 (35)	358 (41.68)	0.4 (0.1, 1.7)	0.237	4 (23.53)	363 (47.39)	0.3 (0.1, 1.2)	0.096
>60	13 (65)	501 (58.32)	Ref.	Ref.	13 (76.47)	403 (52.61)	Ref.	Ref.
**Gender**
Male	9 (45)	357 (41.56)	0.6 (0.2, 1.5)	0.253	9 (52.94)	297 (38.77)	1.5 (0.5, 4.1)	0.47
Female	11 (55)	502 (58.44)	Ref.	Ref.	8 (47.06)	469 (61.23)	Ref.	Ref.
**Occupation**
Employment	5 (25)	96 (11.18)	0.2 (0.1, 1.1)	0.067	2 (11.74)	85 (11.10)	0.8 (0.1, 4.5)	0.803
Unemployment	0	52 (6.05)	12,090,101 (0.0, +∞)	0.998	0	63 (8.22)	17,237,851 (0.0, +∞)	0.997
Retirement	15 (75)	711 (82.77)	Ref.	Ref.	15 (88.23)	618 (80.68)	Ref.	Ref.
**Education**
Primary	3 (15)	521 (60.65)	0.1 (0.0, 0.2)	<0.001	7 (41.18)	497 (64.88)	2.8 (0.8, 9.9)	0.104
Middle	5 (25)	222 (25.84)	0.2 (0.1, 0.6)	0.005	5 (29.41)	167 (21.80)	1.5 (0.4, 5.7)	0.524
High	12 (60)	116 (13.5)	Ref.	Ref.	5 (29.41)	102 (13.32)	Ref.	Ref.
**Type of treatment**
Oral pills	13 (65)	633 (73.69)	0.6 (0.1, 4.8)	0.620	8 (47.06)	445 (58.09)	0 (0.0, +∞)	0.998
Insulin	6 (30)	134 (15.60)	0.3 (0.0, 3.0)	0.316	9 (52.94)	294 (38.38)	0 (0.0, +∞)	0.998
Diet control	1 (5)	92 (10.71)	Ref.	Ref.	0	27 (3.53)	Ref.	Ref.
**Disease duration (years)**
<10	13 (65)	689 (80.21)	0.5 (0.2, 1.4)	0.202	5 (29.41)	339 (44.26)	0.8 (0.3, 2.4)	0.66
≥10	7 (35)	170 (19.79)	Ref.	Ref.	12 (70.59)	427 (55.74)	Ref.	Ref.

*NDR, no diabetic retinopathy; DR, diabetic retinopathy*.

### Attitude

As for the attitude toward DR, more than 90% of the subjects in both groups were concerned about blood glucose and DR complications, and almost all of them were extremely willing to receive more advanced knowledge about DR and cooperate with the follow-up. In the multivariate analysis, those in the NDR subgroup that were unemployed were less likely to have a good attitude (OR = 0.2, *p* = 0.002) (see Table 3).

**Table 3:** Multivariate logistic regression of good attitude in NDR and DR.

	**NDR (*****N*** **=** **879)**				**DR (*****N*** **=** **783)**			
	**Good**	**Poor**	**OR (95%CI)**	***p*-value**	**Good**	**Poor**	**OR (95%CI)**	***p*-value**
**Age (years)**
≤ 60	360 (41.62)	5 (35.71)	3.0 (0.7, 11.9)	0.125	356 (46.41)	11	0.4 (0.1, 1.2)	0.102
>60	505 (58.38)	9 (64.29)	Ref.	Ref.	411 (53.59)	5	Ref.	Ref.
**Gender**
Male	359 (41.50)	7 (50)	0.9 (0.3, 2.8)	0.823	298 (38.85)	8	0.5 (0.2, 1.6)	0.263
Female	506 (58.50)	7 (50)	Ref.	Ref.	469 (61.15)	8	Ref.	Ref.
**Occupation**
Employment	100 (11.56)	1 (7.14)	1.3 (0.2, 10.0)	0.830	86 (11.21)	1	0.4 (0.1, 3.8)	0.452
Unemployment	48 (5.55)	4 (28.57)	0.2 (0.0, 0.5)	0.002	59 (7.69)	4	2.2 (0.6, 8.3)	0.234
Retirement	717 (82.89)	9 (64.29)	Ref.	Ref.	622 (81.10)	11	Ref.	Ref.
**Education**
Primary	513 (59.30)	11 (78.57)	32,047,821 (0.0, +∞)	0.996	490 (63.89)	14	43,510,111 (0.0, +∞)	0.996
Middle	224 (25.90)	3 (21.43)	21,759,974 (0.0, +∞)	0.996	170 (22.16)	2	17,827,840 (0.0, +∞)	0.996
High	128 (14.80)	0	Ref.	Ref.	107 (13.95)	0	Ref.	Ref.
**Type of treatment**
Oral pills	637 (73.64)	9 (64.29)	1.1 (0.1, 9.6)	0.902	442 (57.63)	11	35,307,911(0.0, +∞)	0.998
Insulin	136 (15.72)	4 (28.57)	3.5 (0.4, 33.8)	0.285	298 (38.85)	5	19,224,211(0.0, +∞)	0.998
Diet control	92 (10.64)	1 (7.14)	Ref.	Ref.	27 (3.52)	0	Ref.	Ref.
**Disease duration (years)**
<10	688 (79.54)	14 (100)	0.0 (0.0, +∞)	0.995	338 (44.07)	6	1.8 (0.6, 5.5)	0.282
≥10	177 (20.46)	0	Ref.	Ref.	429 (55.93)	10	Ref.	Ref.

*NDR, no diabetic retinopathy; DR, diabetic retinopathy*.

### Practice

Regarding practice, approximately two thirds (65.39% DR and 67.24% NDR) of the subjects controlled the progression of DR with dietary intervention as necessary. Similarly, three quarters (74.07% DR and 75.54% NDR) of the subjects checked their blood glucose regularly. However, only a quarter of subjects (26.44% DR and 26.62 % NDR) had their eye fundus checked regularly. The risk factors for the DR and NDR groups were evaluated using multivariate logistic regression models, and the results are presented in Table 4. Good practice among the subjects with DR was significantly correlated with age (OR = 0.6, *p* < 0.05), and occupation (OR = 0.4, *p* < 0.05), while good practice among the NDR group was associated with gender (OR = 0.5, *p* < 0.001), educational level (OR = 0.4, *p* < 0.001), and type of treatment (OR = 2.3, OR = 3.1; *p* = 0.001, *p* < 0.001). The remaining factors showed no significant differences.

**Table 4:** Multivariate logistic regression of good practice in NDR and DR.

	**NDR (*****N*** **=** **879)**				**DR (*****N*** **=** **783)**			
	**Good**	**Poor**	**OR (95%CI)**	***p*-value**	**Good**	**Poor**	**OR (95%CI)**	***p*-value**
**Age (years)**
≤ 60	261 (40.03)	104 (45.81)	0.8 (0.6, 1.1)	0.212	264 (43.28)	103 (59.54)	0.6 (0.4, 0.9)	0.023
>60	391 (59.97)	123 (54.19)	Ref.	Ref.	346 (56.72)	70 (40.46)	Ref.	Ref.
**Gender**
Male	250 (38.34)	116 (51.1)	0.5 (0.4, 0.7)	<0.001	229 (37.54)	77 (44.51)	0.8 (0.6, 1.2)	0.344
Female	402 (61.66)	111 (48.9)	Ref.	Ref.	381 (62.46)	96 (55.49)	Ref.	Ref.
**Occupation**
Employment	69 (10.58)	32	1.0 (0.6, 1.7)	0.964	61 (10)	26 (15.03)	0.7 (0.4, 1.2)	0.166
Unemployment	35 (5.37)	17	1.2 (0.6, 2.3)	0.558	37 (6.07)	26 (15.03)	0.4 (0.2, 0.8)	0.005
Retirement	548 (84.05)	178	Ref.	Ref.	512 (83.93)	121 (69.94)	Ref.	Ref.
**Education**
Primary	371 (56.9)	153 (67.4)	0.4 (0.2, 0.6)	<0.001	388 (63.61)	116 (67.05)	1.8 (1.0, 3.3)	0.074
Middle	175 (26.84)	52 (22.91)	0.6 (0.3, 1.0)	0.054	132 (21.64)	40 (23.12)	1.8 (0.9, 3.4)	0.093
High	106 (16.26)	22 (9.69)	Ref.	Ref.	90(14.75)	17(9.83)	Ref.	Ref.
**Type of treatment**
Oral pills	489 (75)	157 (69.16)	2.3 (1.4, 3.6)	0.001	356 (58.36)	97 (56.07)	0.9 (0.4, 2.3)	0.828
Insulin	111 (17.02)	29 (12.78)	3.1 (1.7, 5.6)	<0.001	234 (38.36)	69 (39.88)	0.9 (0.3, 2.3)	0.803
Diet control	52 (7.98)	41 (18.06)	Ref.	Ref.	20(3.28)	7(4.05)	Ref.	Ref.
**Disease duration** ***n*** **(years)**
<10	521 (79.91)	181	1.3 (0.9, 1.9)	0.242	269 (44.10)	75 (43.35)	1.2 (0.8, 1.7)	0.422
≥10	131 (20.09)	46	Ref.	Ref.	341 (55.90)	98 (56.65)	Ref.	Ref.

*NDR, no diabetic retinopathy; DR, diabetic retinopathy*.

## Discussion

This study found that 21.88% of the subjects with DR and 20.98% of those without had a good knowledge of DR, so there was little difference between the two groups. This was consistent with a previous study conducted by Kazi et al. who reported a good knowledge of DR in 20% of subjects (4). However, this figure was less than figures from research in other more developed countries that ranged from 52 to 98% (5–8). Even in the two groups in this study, over 80% of subjects were aware that blood glucose control effectively delays the progression of DR. However, fewer than 7% of subjects in both groups had a good knowledge of the cause, diagnosis, and treatment of DR, indicating this information was virtually unknown to a large majority of them. This finding was in line with two studies conducted by Nihin et al. who also only reported a good knowledge of DR in 9.9% of subjects in South India (9). The lack of knowledge about DR was significantly associated with educational level alone, and the odds of subjects with a primary and middle level of education having a good knowledge of it were 0.1 (0.0, 0.2) and 0.2 (0.1, 0.6) times the odds for those with a high level of education. Similar studies from eastern China (10), Saudi Arabia (11) and India (12, 13) found that those with a lower level of education were less likely to be aware of DR. Since educational attainment is an important measurement of socioeconomic parameters, these people are more likely to be of a lower socioeconomic status and may not be able to afford the expenses for attending eye clinics when their vision is impaired.

Regarding attitude, 94.15% of the DR group and 94.18% of the NDR group said they were concerned, and, thus, there was no difference between the two groups. The results of similar studies showed that over 95% of subjects were concerned about DR (9, 10). In one study, 73.80% of the subjects were aware of the importance of eye checkups, and this was similar to the figure of 75% in an Australian and Saudi Arabia study (10, 14). In our study, there was a statistically significant association between attitude and occupation among the NDR group, the probability of unemployed subjects expressing concern about DR being 0.2 (0.0, 0.5) times lower than that for retired subjects. Unemployed subjects may have more problems in their daily lives and a lower cognitive ability, which may in turn minimize the concerns they have for their health.

Our study indicated that 68.07% of the DR group and 66.92% of the NDR group followed good practice, so there was again no significant difference, and while 74.07% of DR subjects and 75.54% of NDR subjects monitored their blood sugar daily, 80.33 and 82.14% of them, respectively, took their medicine on time. Kazi Rumana found that only 8% of subjects monitored their blood sugar daily and 43% of subjects always took their medicine regularly, far fewer than in our study (6). Previous studies concluded that an annual retinal examination was essential for the early detection of DR (15). According to Murali et al. and AlHargan et al. 82 and 48% of participants, respectively, went for ocular examinations (14, 16), but in both the DR and NDR groups in our study, only 26% of subjects visited the ophthalmologist for regular eye tests, slightly higher than a study in Pakistan (17). This finding may be due to there being no free ophthalmologist consultations for patients with diabetes, yet all hospitals are public in Fushun. In the present study, more than 90% of subjects were willing to cooperate with the follow-up of DR; however, this was not practiced by many subjects. Previous similar study, which were conducted in Myanmar (18), showed that attitudes toward regular examination were favorable but with low compliance. In this study, among the DR subjects, older and retired patients followed better practice, whereas among the NDR subjects, females, those with a high level of education, and those taking oral pills or insulin, followed better practice. The relationship between females and good practice patterns was not unexpected, as it was also reported in other studies, especially in Bangladesh (19–21), and a study of Kuwait (22) also found that practice scores were significantly higher in patients on insulin therapy in comparison with other medications. In China, insulin and antidiabetic pills are only prescribed by an endocrinologist, which means subjects are more likely to follow the doctor's advice. This may also be explained by a change in practice with the progression of the disease. Moreover, a higher level of education might have a positive effect on both good practice and knowledge. This article mentioned that educational attainment and socioeconomic status are closely related. Previous research reported that socioeconomic disparity had a significant association with diabetic knowledge and diabetic management (23). Among the DR group, the older subjects were associated with good practice, whereas younger generations may have more unhealthy dietary patterns or a more sedentary lifestyle, leading to a negative effect on their control of the disease. According to previous studies, the relationship between being young and an increased risk of DR was not typical for our region, but it has been shown to be so elsewhere (24–27). In addition, we found that retired subjects were more likely to follow good practice compared with the unemployed. This could be associated with the fact that retired subjects have more time and have medical insurance to manage the complications of diabetes, and this finding was in agreement with previous studies (28). Practice, particularly regarding life style modification needed to reduce risk of DR and regarding the risk factors for development of eye complications.

In our study, duration of diabetes was not associated to the level of KAP, Tariq Al-Asbali et al. noted no significant association of the duration of diabetes to the level of KAP in Saudi Arabia (29). Perhaps the diabetic patients in our study could be detected early. Four risk factors affect KAP in the NDR group (Gender, Occupation, Education, Type of treatment), yet two risk factors in the DR group (Age, Occupation). The difference in the number of risk factors suggests that a greater need in the NDR group for advocacy and education of different characteristic communities to improve KAP.

Having good knowledge of the disease may be more important in influencing attitudes and practice patterns regarding the disease. As patients' knowledge of the disease increases, their attitudes become positive, ultimately practice become good (30). In this study, the knowledge scores were low, whereas the attitude scores of the subjects were high, indicating a great disparity between knowledge level and attitude level. The gap between knowledge and practice in DR has recently been reported (31). However, the lack of knowledge has always been a major public health concern not only in China, but also in other developing countries(32, 33), so in this context, although the overall knowledge of DR in both the DR and NDR groups was found to be insufficient, this was alarming but not surprising. When the subjects were questioned, they expressed a great thirst for knowledge about DR, but they had no means of obtaining this knowledge, and some subjects did not even know where the eye hospital was. One reason for the lack of knowledge may be poor and uncoordinated health education. Some studies have identified education through health care providers as being the key to improving levels of knowledge (34, 35). Health education measures should be implemented at primary, secondary, and tertiary levels of health care, through the mass media, pamphlets, posters, and DR screening camps, but generally these are not available to patients, which might contribute to their poor levels of knowledge. Another reason is that retinopathy can evolve silently and asymptomatically, so people see no need for any change in their behavior. Therefore, it is necessary to formulate certain strategies and implement measures to ensure good knowledge and positive attitudes can be converted into good practice. Canada through Mobile health education interventions increase KAP in DR individuals and manage chronic diseases and reduce the risk of complications (36).

## Limitations

This study has a few limitations; first, all of the subjects had type 2 diabetes. So, the results cannot be extrapolated to patients with another type of diabetes. Second, the results toward attitude and practice were self-reported by the subjects and not verified by checking with their medical records. Hence, there is a possibility of over-reporting by some of the subjects.

## Conclusion

The study reported a poor level of knowledge of DR but a good attitude and a medium-level of practice in type 2 diabetics with and without DR. The weaker scores were more marked in the young, males, those with only a primary level of education, the unemployed, and those who controlled their diet. Given the reality of the situation in Northeast China, insufficient knowledge in the community with regard to DR is a serious weakness that needs to be addressed. Special outreach and educational efforts are needed to raise awareness in diabetics, in particular those who are at an increased risk of poor KAP, meanwhile there is a greater need to focus on NDR group.

## Data Availability Statement

The original contributions presented in the study are included in the article/supplementary material, further inquiries can be directed to the corresponding author.

## Author Contributions

LW, DL, and CX: conception and design of the research. YW: acquisition of data. Y-BL: analysis and interpretation of the data. K-MF and BZ: statistical analysis. GZ: obtaining financing. J-YQ: writing of the manuscript. D-XZ: critical revision of the manuscript for intellectual content. All authors read and approved the final draft.

## Funding

This study was supported by Liaoning Provincial Natural Science Foundation of China (20170540328).

## Conflict of Interest

The authors declare that the research was conducted in the absence of any commercial or financial relationships that could be construed as a potential conflict of interest.

## Publisher's Note

All claims expressed in this article are solely those of the authors and do not necessarily represent those of their affiliated organizations, or those of the publisher, the editors and the reviewers. Any product that may be evaluated in this article, or claim that may be made by its manufacturer, is not guaranteed or endorsed by the publisher.
